# Sexuality and sexual boundary violations in healthcare organisations: a qualitative focus group study in mental health and disability care in the Netherlands

**DOI:** 10.1136/bmjopen-2025-104483

**Published:** 2026-02-04

**Authors:** Jan-Willem Weenink, Charlotte Kröger, Eva van Baarle

**Affiliations:** 1 Erasmus School of Health Policy & Management (ESHPM), Erasmus University Rotterdam, Rotterdam, The Netherlands; 2 Department of Ethics, Law and Medical Humanities, Amsterdam UMC Location VUmc, Amsterdam, The Netherlands; 3 Netherlands Defence Academy, Breda, The Netherlands

**Keywords:** Disabled Persons, MENTAL HEALTH, Organisation of health services, SEXUAL MEDICINE

## Abstract

**Abstract:**

**Objectives:**

To explore how sexuality and sexual boundary violations are perceived and experienced in healthcare teams and organisations.

**Design:**

Qualitative focus group study.

**Setting:**

Mental health and disability care.

**Participants:**

In total, 56 people participated across 15 focus groups in three healthcare organisations. Participants included client experts (former clients), healthcare professionals such as a psychologist, speech therapist, sexologist and personal coach, team leaders, managers and directors.

**Results:**

We identified 14 different types of situations in which sexuality and sexual boundary violations play a role on four different levels: between clients, between clients and healthcare professionals, between healthcare professionals and on the management level. Situations ranged from attraction and intimacy between clients and/or professionals, promoting sexual health of clients, gut feelings and speaking up, transgressive behaviour from clients and professionals, false accusations and investigations into allegations.

**Conclusions:**

Situations regarding sexuality and sexual boundary violations are varied and complex. They unfold at different levels of interaction within the organisation. To deal with this and come to practical approaches, it is important that clients, professionals and managers engage in reflection and dialogue about their experiences, opinions and perspectives.

**STRENGTHS AND LIMITATIONS OF THIS STUDY**

- A strength was that we held in-depth group discussions with different relevant stakeholders on a client, professional and managerial level.
- In addition, we were able to conduct our research in different healthcare organisations, making our findings less dependent on specific organisational policies and practices.
- A limitation is that mental healthcare and disability care consist of a variety of contexts, client populations and types of care, of which we only covered a selection.
- In addition, we did not specifically explore individual aspects (eg, sexual orientation, religion) as well as broader cultural factors that may impact how people deal with sexuality and sexual boundary violations in practice.

## Introduction

 Mental healthcare and (learning) disability care contexts are known to have a relatively high prevalence of sexual boundary violations (SBV).^1 2^ SBV can be broadly defined as any behaviour or act that transgresses someone’s personal, sexual boundaries.^3 4^ This may include behaviour such as sexual remarks, inappropriate touching and exploitation of power imbalances. At the same time, the two care contexts also include clients that often struggle with sexuality and intimacy.^5 6^ Good sexual health is closely tied to respect for personal boundaries, as sexual health also involves having sexual experiences free of coercion or violations and attention for needs, autonomy, consent and boundaries which can reduce the risk of transgressions while supporting positive sexual well-being.^7^ Facilitating good sexual health while preventing boundary violations presents a moral and practical challenge for healthcare organisations in these care contexts.^8^

In national and organisational policies, sexuality and SBV are generally addressed separately. In legislation, professional codes and institutional regulations, there is a focus on strict rules regarding SBV of healthcare professionals towards clients.^9 10^ For example, intimate contact with a client during or after treatment is prohibited and characterised as sexual violence.^11^ Such strict rules around sexual contact are needed due to the imbalanced power relationship between professionals and clients.^12^ These rules also stem from the immense harm caused by sexual misconduct to clients, whether such misconduct is by a professional or a fellow client.^12^,^14^ Most of the existing research on SBVs is about reacting to incidents of violations, and professionals and organisations find it challenging to adopt and implement preventive measures for SBVs in their work context.^15^,^18^

Facilitating good sexual health of clients has gained increasing attention after a history of more medically oriented views of care in the past.^19^ Intimacy and sexuality might be a part of person-centred care guidelines or a topic that is asked about when a new client enters care.^20^ Previous research shows that this is a challenge, with sexual health needs not being appropriately discussed in mental health and disability care settings, and professionals feeling unprepared or uncomfortable to do so.^21^,^23^

Preventing SBVs and attending to sexual health needs of clients seem two separate topics. Yet, sexual health includes respect for boundaries and both require professionals and organisations to be comfortable and capable of talking about sexual needs, experiences and intimacy.^8 17^ Empowering teams to develop dialogical capabilities to engage with sexuality may open up a space where teams can learn to recognise signals, discuss and address violations at an early stage.^18^ This can be challenging and for some is a taboo, for example, because sexuality is considered a private matter, due to cultural norms or due to a lack of training in how to discuss such issues.^20 24 25^ In addition, the meaning that we might give to sexuality and intimacy is time and context dependent.^26^ To facilitate a meaningful dialogue, it is important to understand in which ways sexuality and SBVs play a role in a specific care context and what challenges involved people encounter. The objective of this study is therefore to explore how sexuality and SBVs are perceived and experienced in healthcare organisations in mental health and (learning) disability care settings and to gain understanding into the complexity of addressing sexuality and SBV in practice.

## Methods

### Setting

This study explores sexuality and SBVs in mental healthcare and disability care organisations, whereby we specifically focus on clients with learning disabilities in the latter. In the Netherlands, healthcare providers are responsible for the quality of care that is provided within their organisation. This includes, for example, adopting person-centred approaches according to relevant guidelines. Guidelines are developed by professional bodies (eg, the nurses association) or associations (eg, mental healthcare association). In addition, there is a specific professional body for sexologists and sexual health advisors. Sexologists are experts on diagnosis and independent treatment of sexual problems and are often psychologists, physicians or psychotherapists with an additional specialised education. Sexual health advisors provide counselling and develop policy on sexual health. They can advise both clients and professionals/organisations.

The healthcare regulator, the Dutch Health and Youth Care Inspectorate, uses existing guidelines to regulate and inspect providers. In addition, providers are mandated to report cases of SBVs by professionals toward clients, as well as sexual violence between clients, to the inspectorate. Individual healthcare professionals are responsible for meeting professional standards and can be individually disciplined in case they have a registered profession (such as physicians, nurses and psychologists). National legislation prohibits intimate contact between a healthcare professional and a client, meaning this can lead to an investigation of the inspectorate or a criminal investigation.^10^

### Study design

We conducted a qualitative focus group study to explore the perspectives and experiences on sexuality and SBV of client experts (former clients), healthcare professionals and managers at three mental healthcare and disability care organisations in the Netherlands. Organisations were purposively sampled based on the care they provided (mental healthcare, disability care) and approached via email. We organised homogenous focus groups to ensure psychological safety and to explore specific experiences within groups of participants. For sessions with client experts with learning disabilities, a personal coach was present to ensure their well-being during and after the session. We opted for smaller size focus groups to allow participants to share their perspective and experiences as extensively as they felt needed. An information letter about the research was shared with participating organisations, and participants were recruited with the help of policy advisors within the participating organisations, who distributed the study information within their organisation and followed up with teams and organisational units. Each participant read and signed an informed consent letter.

### Data collection

We conducted 15 focus groups in three organisations between June and November 2021. Organisation 1 provided both care for people with learning disabilities and mental healthcare, whereas organisations 2 and 3 concerned mental healthcare providers. In total, 56 people participated in the focus groups; 6 client experts, 31 healthcare professionals (such as a psychologist, speech therapist, sexologist and personal coach), 3 team leaders, 10 managers and 6 directors of the healthcare provider. On average, focus groups consisted of four participants, with groups ranging from two participants (ie, directors) to five participants. Participants were sampled based on their ability to provide rich, relevant and diverse perspectives on sexuality and boundary violations, which is also linked to holding different professional functions within both healthcare environments.

The first focus group in each organisation was conducted by two researchers to calibrate the approach, whereas the following sessions were conducted by researchers separately. Each researcher led the data collection in one organisation. A topic list was generated through desk research and consisted of the following topics: (1) preferences and needs of clients regarding sexuality and challenges in addressing these in practice, (2) experiences with preventing and responding to SBV and (3) the role of the organisation and external parties in both.

Ten focus groups were organised online (via Zoom or Teams) due to COVID-19 restrictions at the time, and five were conducted on location at the healthcare provider. An overview of the focus groups can be found in table 1.

**Table 1:** Summary of the focus groups

	Organisation 1	Organisation 2	Organisation 3
Total no of participants	21	18	17
No of focus groups per sector	Five learning disabilities + one mental healthcare	Four mental healthcare	Five mental healthcare
No of participant groups	Four healthcare professionals, one client experts, one managers	One healthcare professional, one client experts, one managers, one directors	Three healthcare professionals, one team leaders, one directors
Leading researcher	CK	EvB	J-WW
Second researcher during first focus group	EvB	J-WW	CK

### Data analysis

Focus groups were audio recorded and transcribed verbatim. Each researcher read and familiarised themselves with the transcripts of ‘their’ organisation and inductively coded relevant sections in the transcripts. In reflexive sessions with the research team, transcripts were discussed, and themes were identified in the data that focused on different kinds of situations of sexuality and SBVs. Based on these themes, researchers went back to the transcripts of their organisation to identify details and examples for each theme. Following this, the research team discussed the themes and examples in additional reflexive sessions, which resulted in combining themes in a final overview that focused on four different thematic levels of interaction.

### Patient and public involvement

Patients or the public were not involved in the design, or conduct, or reporting or dissemination plans of our research.

## Results

From our data, we identified four different thematic levels of interaction where sexuality and SBVs occur as inherently relational phenomena, resulting in a total of 14 different situations on these levels. For each level, we provide specific examples and challenges.

### Level 1: Between clients

Respondents reflected on sexual relationships and situations in which clients desired intimacy. At the same time, situations arose where a client sexually violated boundaries of another client.

#### Situation 1. Sex between clients

Our data showed that healthcare organisations deal with clients’ sexual relationships in different ways. Participants mentioned that in some organisations, it is forbidden for (some) clients to engage in sexual activities, have relationships of any kind, or to sleep in the same room: in these cases, sex or physical intimacy out of sight of professionals is seen as transgressing boundaries. In other organisations, clients are allowed to have sex with each other. There are also organisations where it is unclear what is and what is not allowed. In organisations that prohibit sex between clients, clients sometimes have sex with each other secretly.

On the department, sex or a relationship [between clients] was not allowed. It would mean that one of us would have to go elsewhere. And so, I decided to keep my relationship [with another client] quiet. Because I only have two months left, and as soon as I am gone, I’ll come visit [the other client] (Client)

Clients and professionals mentioned that a consequence of sex being prohibited is that clients do not discuss questions they have about sex and their sexuality with healthcare professionals. Healthcare professionals see this as problematic, as clients might have limited experience or distorted views due to previous experiences with sexual abuse.

#### Situation 2. Boundary-violating behaviour between clients

Both professionals and clients indicated that boundary-violating behaviour between clients occurs. Some situations between clients are clearly unacceptable, such as sexual abuse. But for other types of behaviour, participants mentioned it is not always clear whether this transgresses boundaries. This depends on the rules and practices within a healthcare organisation, and also on the people involved. In disability care in particular, the client population has an impact on the ability to clearly judge whether something is transgressing boundaries, as clients themselves are not always capable of clearly delineating where their boundaries lie. This creates difficult situations for staff, as one professional reflected on a situation where a client had a relationship with another client and laid in bed together.

But I don’t really know, for example, whether she [the client] can clearly indicate her boundaries in this regard. You know, because then her friend will come over… but you don’t really know whether that was her own voluntary choice. Or that it was done under certain pressure and transgressed her boundaries. I find it difficult to assess whether they realize what their boundaries are. (Healthcare professional)

Our data showed that healthcare professionals themselves may also think differently about what is acceptable or not. These different perspectives can be related to their own experiences with sexuality or personal characteristics such as their cultural background or gender and may impact how they judge a situation. For instance, it was mentioned that male professionals may find it harder to talk about sexuality and intimacy with a female client, or the other way around.

### Level 2: Between clients and healthcare professionals

Between professionals and clients, sexuality and boundary violations can play a role in different ways.

#### Situation 3. Paying attention to the sexuality of clients

Healthcare professionals mentioned that talking to clients about sex and intimacy can be difficult. And that it can also be difficult to get someone to explore their sexuality in a healthy and safe way. Especially if a client or professional has had harmful experiences with sex and intimacy in the past. During focus groups, healthcare professionals discussed that experiences of sexual abuse might make it more difficult for a client to be open about sexuality or to be aware of boundaries.

What I also experience, is that clients may have experienced sexual abuse at home and therefore do not really know what the norms and values are surrounding sexuality. (Healthcare professional)

Healthcare professionals themselves might also find it difficult to talk about sexuality with clients. For example, because they do not feel at ease with the topic, or because they lack specific knowledge or skills to address experiences, needs and preferences adequately.

I have a client who has a sex addiction, and I found it quite complicated at first, because she talked about all kinds of things that I personally knew very little about, and she talked in great detail about the swingers’ clubs and the erotic saunas. And well, another 10 steps further. Then at a certain point I was honest and asked: ‘I don’t know much about this, can I ask you some questions because otherwise I won’t be able to help’. (Healthcare professional)

At the same time, clients might find it difficult to bring up issues regarding sexual desires themselves, even when they have a clear need for it. Having a healthcare professional that feels comfortable to discuss sexual health needs might lower the threshold to express their wishes.

At that time, I was on medication and had an erection problem. That it [sexuality] was a question and suddenly we could discuss this, I really felt much less alone and seen… That really made me feel more human, just because of that issue. On the other hand, I don't think I would be able to raise this issue myself (Client)

#### Situation 4. A healthcare professional is attracted to a client

Healthcare professionals mentioned that sexual temptations, attraction and relationships can arise at work, and as such a healthcare professional may develop feelings of attraction towards a client. Although professionals acknowledged the possibility of this during focus groups, they were reluctant to talk about developing such feelings themselves, and the ones who did talk about it indicated that they were able to regulate such feelings without acting on them.

Well, before I worked here… I fell in love with everyone. For me, being in love is also being able to see the beautiful qualities someone has. Falling for a beautiful laugh, great comment, wit, a warmth, you know that can just happen. I'm not going to say that to the client or to a colleague. I've learned over the years to channel this differently. I can say to people ‘do you realize how nice you are’, you know, positive feedback. I say that to men and women. (Healthcare professional)

#### Situation 5. Judging a signal of sexual abuse from a client

Professionals mentioned that if a client reports to have experienced sexually transgressive behaviour, it can sometimes be difficult to properly assess whether or not this has happened. A complicating factor that was mentioned is that stigma related to the client’s diagnosis sometimes leads to a client not being believed when speaking up about transgressive behaviour. In addition, situations occur where someone else suspects that a client and healthcare professional have sexual contact, but both deny this.

Well, what first happened was that rumours started going around, then the supervisor had a conversation with the client and the colleague. They denied it, that was it. And I think a month later, rumours came up again, the same ones with a new group. So, with other clients in the group and they gave the same signals. (Healthcare professional)

#### Situation 6. The client transgresses the boundaries of a healthcare professional

Participants mentioned that people’s personal boundaries differ, and not everyone experiences the same situations as a boundary violation. This applies to clients and to professionals. Sometimes sexually transgressive behaviour is a grey area in which it is not clear whether behaviour is transgressing boundaries or opinions differ. Professionals also highlighted that it is important to recognise that some healthcare professionals find it easier to deal with transgressive behaviour than others.

We had a colleague who was stalked by a client and received letters that were sexually explicit. We had a client made a comment to a young colleague about how nice she looked. A male colleague can easily make fun of that. While a female colleague might find it annoying that this is said. You also must know each other’s boundaries. I may find it funny, but if a colleague doesn't find it funny, he should point that out. (Healthcare professional)

#### Situation 7. False accusations of SBVs

Professionals raised experiences with accusations that were false or unfounded. In contrast to talking about developing sexual feelings for clients, professionals found it easier to talk about their experiences with wrongful accusations. When the topic of sexual misconduct of a professional was raised, often examples of false accusations were given. These false accusations can in themselves have a serious impact, both on the (emotional well-being of the) professional that is being accused but also on the team.

We had a situation of a male colleague who had to go to a female client’s room. And I think he had to shower her. That is always without physical contact, and with a lot of respect. But then she, I think also because of an experience, made it up that he had touched or assaulted her in some way. While he swore that he had not been near her. This had quite some consequences, because we had to take that very seriously. While we as a team also realized that this followed from her mental illness, from trauma or a psychotic image. We really sat around the table with the client, the colleague, the manager and I think someone else too. To really work on it so that the client felt heard and could see that something was being done about it, so to speak. But deep down we knew that it was not a serious allegation. (Healthcare professional)

In talking about unwarranted accusations, both male and female healthcare professionals highlighted that especially (heterosexual) male healthcare providers are vulnerable to false allegations, with some avoiding one-on-one contact with female clients altogether.

#### Situation 8. How close do you get to your client?

Professionals mentioned that different clients have different needs for intimacy, and that the same goes for healthcare professionals. Sometimes a client wants an arm around their shoulder, yet not all healthcare professionals feel comfortable in giving one. And even if a healthcare professional does feel comfortable, the question is how to determine how close you can get to a client. Respondents indicated that they experience a shift in time in what is normal and acceptable and what is not, partly driven by a change in societal and professional norms.

When I started working in mental healthcare, you could still put your arm around someone’s shoulder, so to speak, as a kind of comfort if someone was sad. And you know, I'm also someone who likes to make physical contact, without transgressing any boundaries, but I do put an arm around someone. Like saying, ‘come on, keep it up today’. And then we had a period in which you were trained as a nurse with the idea of professional distance. At a certain moment it was 'not done’ to support someone in that way, so to speak, and then you really feel a kind of coldness in the contact. (Healthcare professional)

### Level 3: Between healthcare professionals

Sexuality also plays a role between healthcare professionals and could relate to the behaviour of a colleague towards a client or behaviour between colleagues themselves.

#### Situation 9. Sexuality between colleagues

During focus groups, healthcare professionals discussed that colleagues sometimes develop feelings for each other that might develop into an intimate relationship. Different protocols and views from healthcare professionals exist on relationships between colleagues and whether this is acceptable. In addition, transgressive behaviour happens between colleagues. Professionals mentioned that it is not easy to speak up or report transgressive behaviour within their team.

Reporting something about a client who is behaving inappropriately towards a healthcare provider is not too difficult. But among colleagues, I think, in all teams, there is a high threshold to report, and in clinical teams even higher. People don't do that so quickly. You have to challenge people, create a safe environment where they can report things about their colleagues. That is incredibly complicated. To be open, transparent. (Healthcare professional)

#### Situation 10. Discussing sexuality and transgressive behaviour in teams

Professionals recognised the importance of discussing experiences and views on sexuality and transgressive behaviour with each other. Some had experience with talking about these aspects in their team, though most respondents indicated that they did not explicitly discuss their own norms and values regarding these issues. The need for a safe environment to do so was mentioned, and how factors such as hierarchy and team size might create barriers to having such conversations.

I think when I look at us, that hierarchy and the number of people who are sitting at the table matter. Because very often it is said for the sake of appearances that we have a very safe team and can discuss everything with each other. But in the meantime, a lot is happening in all kinds of areas, also out of sight, in the corridors as we call it. So, I think it is also a bit of personal task of colleagues with whom you work closely to occasionally bring up [sexuality and intimacy] for discussion. (Healthcare professional).

#### Situation 11. Gut feelings

Healthcare professionals mentioned that they sometimes might have a gut feeling about something that does not seem right between a colleague and a client, or between colleagues. Something seems wrong, but if you’re not sure or have any evidence, it can be difficult to discuss such gut feelings with colleagues. Professionals then sometimes feel they might do harm by speaking up but being wrong and damaging the colleague, or by not speaking up and letting a transgressive and harmful situation unfold and continue.

At some point you get a gut feeling that you think: I don't have a good feeling about this. Such a bad feeling. Then it often takes a long time before you can put your finger on it. It’s the same when you suspect someone of theft. Those are the kinds of things that make you think: I can't just tell that person that he’s stealing or that he’s displaying transgressive behaviour. Then you really have to know what you're talking about. Because you can also really damage that employee if it’s not true. (Healthcare professional)

#### Situation 12. An investigation into sexually transgressive behaviour of a colleague

Respondents who had experienced investigations into alleged transgressive behaviour by a healthcare professional towards a client described the process as tough, lengthy and emotionally demanding. They noted that even when the colleague is ultimately found not guilty, the investigation itself can erode trust within the team – not because of the false accusation, but because of the impact of the investigation process.

I can still remember how miserable some colleagues felt. Because they were really convinced of [the colleague’s innocence] and they literally put their own skin on the line to defend that colleague. And I can still remember how awful they felt. They really felt cheated. Yes, that really stuck with me. And what the organisation offered then was too little, I think. They paid attention to it during one meeting. (Healthcare professional)

Managers and directors also reflected on the challenging aspect of how to learn from investigations into transgressive behaviour and to rebuild trust, especially because these investigations bring out a lot of emotions among people, such as loyalty conflicts and feeling betrayed.

### Level 4: Management

On a management and organisational level, dealing with sexuality, intimacy and transgressive behaviour involves, among other things, drawing up protocols and guidelines. And initiating and responding to reports and investigations into sexually transgressive behaviour.

#### Situation 13. Sexuality on the agenda

Healthcare professionals indicated that directors and managers play a crucial role in the organisation when it comes to adequate policy and putting sexuality and transgressive behaviour on the agenda, as those in a position of leadership should ensure that sexuality can be discussed in the organisation. Most professionals and managers indicated that they were not aware of clear policies on sexuality in their organisation. They stated the importance of policy in addressing sexuality adequately, while at the same time also recognised that policy by itself is insufficient.

That you make policy on certain issues. I think we can still make progress on that, because whether healthcare workers ever talk about [sexuality and sexual boundaries]; it is sometimes discussed within the team, but whether we really have a policy on that, I wouldn’t even know for each of our locations. And [policy] is what could give you support as an employee. (Manager)

#### Situation 14. Conducting an investigation into sexually transgressive behaviour

Respondents mentioned that internal and external investigations into sexually transgressive behaviour can have an enormous impact. Most importantly to the person whose boundaries are violated, but also to the person that reports the behaviour, the team, managers and the executive team. Managers and directors mentioned it is difficult to determine how to guarantee privacy of involved people on the one hand and, on the other hand, communicate properly about a lengthy investigation to employees in the organisation. Often, people in the organisation realise something is up and rumours start going around. Managers indicated that they struggle with deciding what to communicate and at what time.

I also recognize what my colleague says, the tension between what you communicate and what you do not communicate. How do you communicate to employees who are your colleagues, what do you tell? And something I myself often have questions about, certainly regarding other incidents within the organization: how can we learn from the incidents? In a way that you can protect the people it concerns, but at the same time you want to learn something from the incident as an organization. And that is a bit of my search: when are you actually doing well as an organization? (Manager)

## Discussion

Our findings demonstrate that clients, professionals and managers face various situations involving sexuality and transgressive behaviour in practice, which unfold at different levels of interaction within the organisation. When promoting sexual health, creating space for intimacy or preventing SBVs, it is important to be specific about what this entails. Our results highlight the multitude of aspects that must be considered to address sexuality and SBV adequately. In this section, we discuss why reflection and dialogue are critical, and why the complexity on different organisational levels and interactions between these levels must be acknowledged.

### Reflection and dialogue to deal with varied and complex situations of sexuality and SBV

The varied experiences and perspectives of respondents indicate the importance of clear rules and policies about what is acceptable and what is not (content) and how to deal well with sexuality, intimacy and SBV investigations (process). As literature suggests, it became evident that it is as important to facilitate reflection and dialogue about sexuality and SBV in practice.^21^,^23^ Respondents at times differed in their views of acceptable behaviour or how to attend to clients’ sexual needs. Empowering teams to develop reflexive and dialogical capabilities can enhance early recognition and response to violations and help establish shared agreements while accommodating differing perspectives.^8 18 27^

Embedding meaningful dialogue requires two things. First, professionals, clients and managers need the skills to engage in dialogue. This involves the ability to discuss sexuality and sexual needs openly, which our study showed to be challenging for both professionals and clients.^28^ It also refers to speaking up about concerns; bystander-effect training could help people to intervene when boundaries are violated.^29^ Additionally, normalising conversations around sexuality and boundaries might translate into less shyness to act on them.^8^ It is important to support clients in developing skills to talk about these subjects, especially in disability care and mental healthcare, where they often struggle with this and face stigma.^30^

Second, dialogue needs to be structurally embedded in the processes and practices of teams and their organisations.^15 31 32^ Talking about sexuality and SBV requires vulnerability, as you need to be open about your own personal values and beliefs.^18^ Professionals and clients who are in a hierarchical or dependent relationship might refrain from speaking up or asking questions because they fear negative consequences, for example, by being judged or punished by a superior. Managers can have a leading role by openly discussing sexuality and showing vulnerability, thus setting the tone for the organisation.^33^,^35^

### Acknowledging complexity and interactions between organisational layers

Our study identified 14 different situations of sexuality and SBV across organisational layers, demonstrating the complexity on each. Clients struggled with their sexual needs but felt uncomfortable talking about this, sometimes fearing possible consequences for their relationship with another client. Professionals wanted to deal with client needs but sometimes felt inadequate and struggled to voice concerns, while wanting to protect clients against transgressive behaviour. Different moral perspectives between clients and professionals on sexual health and intimacy can make conversations even harder.^36^ Especially when there are different gender identities and sexual preferences involved.^37 38^ Managers had to deal with complaints and investigations into SBV, yet struggled with communicating about and learning from such investigations.

A perhaps logical response is to develop distinct practical approaches per stakeholder group, but this would ignore the interactions between these layers. Teams might be influenced by top-down managerial decisions, practices and behaviour.^39^ If management does not signal sexuality and SBV as important themes, professionals likely won’t pay them much attention. At the same time, clients and professionals might impact policies and practices on a management level through co-creative, bottom-up approaches. Recognising that discussing and addressing clients’ sexual needs is challenging but beneficial may encourage organisational support, via policies, training and education. Acknowledging the interaction between layers could open up the discussion about what the organisation—on different levels—runs into. And it could contribute to making sexuality and SBV a shared responsibility for everyone involved.

### Strengths and limitations

A key strength is the in-depth group discussions with different stakeholders, providing a broad picture of sexuality and SBVs in healthcare organisations. Conducting our research in different healthcare organisations makes our findings less dependent on specific organisational policies and practices.

The study also has some limitations. First, most focus groups were organised online, primarily due to COVID-19 restrictions. This could have impacted openness, though we did not notice notable differences between the online and physical groups. Second, mental healthcare and disability care consist of a variety of contexts, client populations and types of care, which we did not all cover. For disability care, for example, we included clients with mild learning and intellectual disabilities. Several individual aspects such as sexual orientation and religion may impact people’s experiences, and they might also differ between national contexts. We must therefore be careful in generalising findings to broader settings. Future studies could explore these aspects in more detail.

### Conclusion

Our study shows that in practice, sexuality and SBV are multifaceted. Clients, professionals and managers should engage in reflection and dialogue about their experiences, opinions and perspectives to deal with these varied and complex situations. The situations and challenges identified in this study can help organisations in developing context-specific policies and practices.

## Data Availability

No data are available.
