# Supplementary material for: Sexuality and sexual boundary violations in healthcare organisations: a qualitative focus group study in mental health and disability care in the Netherlands
Source: BMJ Open. 2026 Feb 4;16(2):e104483. doi: 10.1136/bmjopen-2025-104483 (PMC12878329; doi:10.1136/bmjopen-2025-104483)
Supplement: Supplementary file 1 [file bmjopen-2025-104483.reviewer_comments.pdf]

PEER REVIEW HISTORY

BMJ Open publishes all reviews undertaken for accepted manuscripts. Reviewers are asked to complete a checklist review form and are provided with free text boxes to elaborate on their assessment. These free text comments are reproduced below.

ARTICLE DETAILS

Title (Provisional)

Sexuality and sexual boundary violations in healthcare organisations: a qualitative focus group study in mental health and disability care

Authors

Weenink, Jan-Willem; Kröger, Charlotte; van Baarle, Eva

VERSION 1 - REVIEW

**Reviewer**	**1**
**Name**	**Vesentini, Lara**
**Affiliation**	**Vrije Universiteit Brussel, Mental Health and Wellbeing Research Group**
**Date**	**25-Jun-2025**
**COI**	**None**

Thank you for the opportunity to read this paper on an important topic that is understudied.

Although the research is well conducted and results are interesting, I think the paper needs a major revision. The text in the paper should be better structured.

Introduction.

Introduction can be better structured. To do so, it should be first of all crystal clear what the research objective is. Is it to identify the challenges people encounter? To identify the ways sexuality and SBV is present? Is it to identify different levels of interaction? To identify different situations?

If the main focus is on the situations in different organizational levels, the introduction should focus more on that aspect. Furthermore, it should be more defined what SBV is, and how sexual health and SBV are related to one another.

Method

Again, the method section can be better structured.

I would suggest making a subheading ‘study population’, where you first describe the organizations, and then the different types of participants. For example, explain what client

experts are (e.g., it is mentioned in ‘study design’, but only in the paragraph about data collection you make clear that client experts are former clients). Also, I’m still not sure if disabled care is about physically or mentally disabled care.

Als a subheading recruitment is needed. Explain more in detail how participants were recruited via their organization. Was there a difference between clients, managers, health care professionals? Further I would like to know how many persons participated from each subcategory, i.e., how many former clients in total, how many managers/directors, and how many healthcare professionals.

The first focus groups in each organization were conducted by two researchers, but there were in total three researchers, so I do not understand how they did it.

The subheadings in table 1 are not clear. For example, replace ‘sector’ into ‘No. of focus groups per sector’. Idem Participant groups. Furthermore, I would suggest moving the row ‘total no of participants’ to the top, because then the rows below all refer to the number of focus groups.

The description of the data analysis is rather brief. Was the data thematically analyzed? Did they work inductively or deductively? If the research objective was to identify different situations at different organizational levels, it is partly deductive, as they already know which levels there are, I suppose.

Results

I’m not convinced the figure is an added value.

Throughout the whole results section it should be clearer if the statements/opinions are from the participants or if it’s theory. For example, as described in situation 3: “Particularly in mental health and disability care, the prevalence of people…” Or in situation 6: "Peoples’s personal boundaries differ, and not everybody …”. Overall, I would suggest formulating more sentences such as ‘Participants mentioned that …’. Or in situation 4: However, intimate relationships between caregivers and clients are prohibited due to the inherent power imbalance…” . Also, make clearer if it is just the opinion of healthcare providers or also of clients, and/or managers.

Only very few citations of clients are used. Why is that?

Used consequently the same terminology: clients instead of patients, healthcare professionals instead of caregivers or healthcare providers, transgressive behavior instead of crossing behavior.

In situation 6 it is stated that ‘And it is important to recognize that some healthcare professionals find it easier to deal with boundary crossing behavior than others’. Do participants indicate it is important or is this the interpretation of the author. Interpretations should not be in the results section.

I would suggest leaving out situation 9, as it is not relevant within the scope of this paper. If the authors think this is relevant, it should be also mentioned in the introduction.

The difference between situation 12 and situation 7 is not clear to me.

Overall, I find the four different levels: between clients, between clients and health care professionals, between healthcare professionals and management rather confusing. For me personally, I see following themes:

As a client dealing with sexuality and SBV

-Doing it secretly (situation 1)

-Difficult to discuss (Situation 3)

As a Healthcare Professional dealing with

-Attraction to a client (situation 4)

-a report of SBV, by a client (Situation 5)

- client transgress boundaries (Situation 6)

- being falsely accused (Situation 7)

- suspicion of SBV (Situation 11)

-complaint of SBV (situation 12)

Other related difficulties / needs for healthcare professionals

-Delineate boundaries for clients (situation 2)

-Discussing sexuality with clients (situation 3)

-Discussing sexuality in teams (situation 10)

-Distance to client (Situation 8)

Difficulties and challenges for management

-Healthcare organizations deal with sexual relationships in different ways (situation 1)

-Being not aware of clear polices (situation 13)

-Difficult to communicate when there is an investigation (situation 14)

Discussion

The discussion is too long and should be shortened. Because the research objective is not clear, also the discussion is not that clear. Overall, I read some recommendations like how to improve dialogue, but reviewing the results, comparing them with other similar studies, and explaining results are lacking.

Reviewer 2
Name Muller, Madeleine
Affiliation Walter Sisulu University, Family Medicine
Date 26-Aug-2025
COI None

This is a great paper, with an important research question and solid methodology. Most of my input is around the structuring of the information – keeping results and discussion completely separate. A couple of sections on the methodology need more information.

Sexuality and sexual boundary violations in healthcare organisations: a qualitative focus group study in mental health and disability care

This is a great paper, with an important research question and solid methodology. Most of my input is around the structuring of the information – keeping results and discussion completely separate. A couple of sections on the methodology need more information.

Page 2:
Line 16: Setting
Sexuality and sexual boundary violations in healthcare organisations: a qualitative focus group study in mental health and disability care

Page 3
Line 6: Please add a definition of Sexual boundary violations.

Line 17 for grammatical clarity: change from ‘follow from the’ to ‘are needed due to’

Page 4
Line 6: This sentence is unclear and maybe needs an addition.

Line 12 Perhaps replace ‘specialized’ with 'are experts on'

Line 48: Add a comment on the total sample size - how many potential participants did you do your purposeful sampling from.

Page 5:
Line 51: This section needs additional comment on the type of thematic analysis that was conducted. How did you come to themes? Was data first coded? How many researchers were involved in the analysis and how did you interact (e.g. parallel coding or analysing together).

Page 6

Line 12: You used the word 'themes' in your data anlysis but here you are talking about 4 levels - perhaps this was more of a content analysis? Just make sure it is alligned.

Line 51: Emphasize that this is what your data showed - this is what was reported in the groups.

Page 7
Line 43: Indicate that this is a finding from the data at the beginning of the sentence.

Line 58: Was this shown in the data - focus on what came out of your data. Leave discussion for later.

Page 9
Page 16: if this is a finding from the data you can remove the word 'might'

Page 37: This whole section sounds like an interpretation / recommendation. Under the data - focus on reporting what you found. Move any interpretations / recommendations to the discussion.

Page 10
Page 39: Be careful not to put 'discussion points' in your data sections - it happens here and there. Focus on reporting what the data showed you. Leave the interpretations for the discussion.

Page 11
Line 17: This sounds more like a discussion / introduction to this topic- stick with what the data shows. You can add this to discussion later.

Page 12:
Line 3: Rephrase to show that this is what the data showed (like the previous paragraph)

Page 13:
Line 23 Make sure your paragraph reflect the data. If this is what your participants said - then make that clear.

Line 46L Great discussion and reflection. If there are discussion points that I flagged in your results, you can add these in if it feels important.

** The reviewer provided a marked copy with additional comments. Please contact the publisher for full details.**

VERSION 1 - AUTHOR RESPONSE

Reviewer 1

**Introduction**	
Introduction can be better structured. To do so, it should be first of all crystal clear what	Thank you for this valid point. We have elaborated on the objective as ‘to explore

the research objective is. Is it to identify the challenges people encounter? To identify the ways sexuality and SBV is present? Is it to identify different levels of interaction? To identify different situations? If the main focus is on the situations in different organizational levels, the introduction should focus more on that aspect.	how sexuality and sexual boundary violations are perceived and experienced in healthcare teams and organisations'. We have adjusted the text in the abstract and introduction. The main focus of the study is not on different organisational levels. This is something that followed from our findings and analysis, and we therefore do not focus on it in the introduction but instead discuss these levels and their interaction in the discussion of the paper.
Furthermore, it should be more defined what SBV is, and how sexual health and SBV are related to one another.	Thank you for this comment. We have included a definition of SBV and some examples for illustration in the first paragraph of the introduction. In the introduction, we mention the relatedness of sexual health and SBV as good sexual health also involves awareness of boundaries and both require openness and comfort with a sensitive topic such as sexuality and intimacy, and we elaborate how attention and competence with sexuality might also aid adequate dealing with SBV. We have added a sentence to clarify this relationship further.
**Method**	
I would suggest making a subheading 'study population', where you first describe the organizations, and then the different types of participants. For example, explain what client experts are (e.g., it is mentioned in 'study design', but only in the paragraph about data collection you make clear that client experts are former clients). Also, I'm still not sure if disabled care is about physically or mentally disabled care.	Thank you for this suggestion. We have integrated the information on the study population and recruitment in the study design section that client experts are former clients with learning disabilities and have also specified in the first sentence of the setting section that we focus on care for people with learning disabilities in this article.
Also a subheading recruitment is needed. Explain more in detail how participants were recruited via their organization. Was there a difference between clients, managers, health care professionals? Further I would like to know how many persons participated from each subcategory, i.e., how many former clients in total, how many managers/directors, and how many healthcare professionals.	Thank you for this suggestion. We have integrated the information on the study population and recruitment in the study design section: *'Individual participants were recruited with help of policy advisors within the participating organisations, who distributed the study information within their organisation and followed up with teams and organisational units.'*<br>We also have included the total number of client experts, healthcare professionals,

	team leaders, managers and directors in the data collection paragraph: *'In total, 56 people participated in the focus groups; 6 client experts, 31 healthcare professionals (such as a psychologist, speech therapist, sexologist and personal coach), 3 team leaders, 10 managers and 6 directors of the healthcare provider. On average, focus groups consisted of 4 participants, with groups ranging from 2 participants (i.e. directors) to 5 participants'*. We also touch upon differences between these groups. Moreover, we opted not to make a separate paragraph as we felt putting this information in one paragraph and taking it out of others would impact the overall structure of the methods section.
The first focus groups in each organization were conducted by two researchers, but there were in total three researchers, so I do not understand how they did it.	We agree that we should clarify this. Each first focus group in each organisation was conducted by two of the three researchers with three unique couples, so each researcher conducted two out of three first focus groups. Organisation 1 was done by CK and EvB, in organisation 2 by EvB and JWW, and in organisation 3 by JWW and CK. We have rephrased the sentence and have added to Table 1 which second researcher was present during the first focus group.
The subheadings in table 1 are not clear. For example, replace 'sector' into 'No. of focus groups per sector'. Idem Participant groups. Furthermore, I would suggest moving the row 'total no of participants' to the top, because then the rows below all refer to the number of focus groups.	Thank you for this suggestion. We agree and we have moved the row 'total no. of participants' to the top, have merged the sector and number of focus groups rows into one (number of focus groups per sector), and have adjusted the participant heading into 'no. of participant groups'.
The description of the data analysis is rather brief. Was the data thematically analyzed? Did they work inductively or deductively? If the research objective was to identify different situations at different organizational levels, it is partly deductive, as they already know which levels there are, I suppose.	Thank you for this. We agree and we have added more detail to the data analysis paragraph, elaborating on how transcripts were inductively coded, and how reflexive sessions with the research team led to the final themes. No predetermined framework was used; the different organisational levels followed from our analysis and was not an objective by itself.<br>We have adjusted the text as follows:

	'Focus groups were audio recorded and transcribed verbatim. Each researcher read and familiarised themselves with the transcripts of 'their' organisation and inductively coded relevant sections in the transcripts. In reflexive sessions with the research team, transcripts were discussed, and themes were identified in the data that focused on different kind of situations of sexuality and sexual boundary violations. Based on these themes, researchers went back to the transcripts of their organisation to identify details and examples for each theme. Following this, the research team discussed the themes and examples in additional reflexive sessions, which resulted in combining themes in a final overview that focused on four different thematic levels of interaction.'
**Results**	
I'm not convinced the figure is an added value.	Thank you for this comment. As suggested, we have removed the figure from the manuscript.
Throughout the whole results section it should be clearer if the statements/opinions are from the participants or if it's theory. For example, as described in situation 3: "Particularly in mental health and disability care, the prevalence of people…" Or in situation 6: "Peoples's personal boundaries differ, and not everybody …". Overall, I would suggest formulating more sentences such as 'Participants mentioned that …'. Or in situation 4: However, intimate relationships between caregivers and clients are prohibited due to the inherent power imbalance…" . Also, make clearer if it is just the opinion of healthcare providers or also of clients, and/or managers.	We agree and we have gone through the findings, rephrased statements and opinions from participants, removed any interpretation from our side, and have highlighted which respondent group(s) mentioned a particular perspective or opinion.
Only very few citations of clients are used. Why is that?	Thanks for this question. This follows from two things. First, from our data we identified four different thematic levels of interaction where sexuality and SBVs occur as inherently relational phenomena, resulting in four different levels: perspectives and experiences of clients were most relevant in the first two levels. In addition, only two

	focus groups were held with clients, and we've tried to use data from different focus groups to illustrate our findings throughout the manuscript.
Used consequently the same terminology: clients instead of patients, healthcare professionals instead of caregivers or healthcare providers, transgressive behavior instead of crossing behavior.	We have gone through the manuscript and have adjusted the language, so it is used consequently, i.e. clients, healthcare professionals, and transgressive behaviour. The only part where we still mention patient is in the mandated 'patient and public involvement' section.
In situation 6 it is stated that 'And it is important to recognize that some healthcare professionals find it easier to deal with boundary crossing behavior than others'. Do participants indicate it is important or is this the interpretation of the author. Interpretations should not be in the results section.	Thank you for this suggestion. We have rephrased this sentence to clarify that this is what professionals mentioned during focus groups.
I would suggest leaving out situation 9, as it is not relevant within the scope of this paper. If the authors think this is relevant, it should be also mentioned in the introduction.	We have clarified the scope of the paper in the introduction in which we take a broader view on SBV, which now clarifies the relevance of situation 9 for the scope if this paper.
The difference between situation 12 and situation 7 is not clear to me.	Thank you for this question. We feel these are two separate issues, that in practice often are linked. Situation 7 focuses on false accusations and the impact they have. Situation 12 is about investigations into transgressive behaviour and details the impact and challenges of the investigative procedure. In practice, false accusations might lead to investigative procedures, but they do not necessarily always do (e.g. because it is decided not to launch a formal investigation). We have clarified this difference in the description of the situations.
Overall, I find the four different levels: between clients, between clients and health care professionals, between healthcare professionals and management rather confusing. For me personally, I see following themes:<br><br>As a client dealing with sexuality and SBV<br>-Doing it secretly (situation 1)	Thank you for this suggestion. As stated above we have focused on the diverse ways sexuality and sexual boundary violations are present in healthcare teams and organisations. In the methods section, we have now clarified how the four levels followed from our thematic analysis. They provide structured insight in diverse scope and complexity of sexuality and SBV. We

-Difficult to discuss (Situation 3)<br><br>As a Healthcare Professional dealing with<br>-Attraction to a client (situation 4)<br>-a report of SBV, by a client (Situation 5)<br>- client transgress boundaries (Situation 6)<br>- being falsely accused (Situation 7)<br>- suspicion of SBV (Situation 11)<br>-complaint of SBV (situation 12)<br><br>Other related difficulties / needs for healthcare professionals<br>-Delineate boundaries for clients (situation 2)<br>-Discussing sexuality with clients (situation 3)<br>-Discussing sexuality in teams (situation 10)<br>-Distance to client (Situation 8)<br>Difficulties and challenges for management<br>-Healthcare organizations deal with sexual relationships in different ways (situation 1)<br>-Being not aware of clear polices (situation 13)<br>-Difficult to communicate when there is an investigation (situation 14)	feel it is important to acknowledge that sexuality and boundary violations are inherently relational phenomena which involve interactions between different stakeholders. This goes beyond individual client or professional experiences with either sexuality or SBV.
**Discussion**	
The discussion is too long and should be shortened. Because the research objective is not clear, also the discussion is not that clear. Overall, I read some recommendations like how to improve dialogue, but reviewing the results, comparing them with other similar studies, and explaining results are lacking.	Thank you for this suggestion. As we have clarified the research objective, we have also worked on better aligning the introduction and discussion section. Facilitating good sexual health of clients has gained increasing attention, however, previous research shows that this is a challenge, with sexual health needs not being appropriately discussed in mental health and disability care settings, and professionals feeling unprepared or uncomfortable to do so. We argue that to facilitate a meaningful dialogue, it is important to understand in which ways sexuality and SBVs play a role in a specific care context and what challenges involved people encounter. In the discussion, we therefore focus on how our findings may foster reflection and dialogue to deal with these situations, and why it is important to acknowledge the complexity on different

	organizational levels and interactions between these levels when approaching sexuality, intimacy and boundary violations in healthcare organisations.<br><br>We agree that the discussion section is too long and have shortened it considerably.

Reviewer 2
This is a great paper, with an important research question and solid methodology. Most of my input is around the structuring of the information – keeping results and discussion completely separate. A couple of sections on the methodology need more information

Page 2:<br>Line 16: Setting<br>Sexuality and sexual boundary violations in healthcare organisations: a qualitative focus group study in mental health and disability care	We have added an introductory sentence to the Setting section, where we repeat that this study focusses on exploring sexuality and sexual boundary violations in mental health and disability care organisations.
Page 3<br>Line 6: Please add a definition of Sexual boundary violations.<br>Line 17 for grammatical clarity: change from 'follow from the' to 'are needed due to'	We have included a definition of SBV and some examples for illustration in the first paragraph of the introduction. We have also made the suggested textual change.
Page 4<br>Line 6: This sentence is unclear and maybe needs an addition.<br>Line 12 Perhaps replace 'specialized' with 'are experts on'<br>Line 48: Add a comment on the total sample size - how many potential participants did you do your purposeful sampling from.	We have tried to clarify the sentence (i.e. *In the Netherlands, healthcare providers are responsible for the quality of care that is provided within their organisation*).<br>We've changed 'specialized' to 'are experts on'.<br>We have also added information on the total sample size. We purposefully sampled organisations based on care context. However, our participant sampling did not occur based on the total sample size of healthcare professionals within the organisations, but on information richness and specific content criteria. i.e. we selected participants based on their ability to provide rich, relevant and diverse perspectives on the issue, which is also linked to them holding different professional functions within both healthcare environments. We list the job positions in the data collection section and added a sentence to explain this approach.
Page 5:	We have added more detail to the data analysis paragraph, elaborating on how

Line 51: This section needs additional comment on the type of thematic analysis that was conducted. How did you come to themes? Was data first coded? How many researchers were involved in the analysis and how did you interact (e.g. parallel coding or analysing together).	transcripts were inductively coded, and how reflexive sessions with the research team led to the final themes:<br><br>*'Focus groups were audio recorded and transcribed verbatim. Each researcher read and familiarised themselves with the transcripts of 'their' organisation and inductively coded relevant sections in the transcripts. In reflexive sessions with the research team, transcripts were discussed, and themes were identified in the data that focused on different kind of situations of sexuality and SBVs. Based on these themes, researchers went back to the transcripts of their organisation to identify details and examples for each theme. Following this, the research team discussed the themes and examples in additional reflexive sessions, which resulted in combining themes in a final overview that focused on four different thematic levels of interaction.'*
Page 6<br>Line 12: You used the word 'themes' in your data analysis but here you are talking about 4<br>levels - perhaps this was more of a content analysis? Just make sure it is aligned.<br>Line 51: Emphasize that this is what your data showed - this is what was reported in the<br>groups.	We have rephrased the sentence, so it is clear this followed from our data.
Page 7<br>Line 43: Indicate that this is a finding from the data at the beginning of the sentence.<br>Line 58: Was this shown in the data - focus on what came out of your data. Leave discussion for later.	We have rephrased both sentences, so it is clear these findings followed from our data.
Page 9<br>Page 16: if this is a finding from the data you can remove the word 'might'<br>Page 37: This whole section sounds like an interpretation / recommendation. Under the data - focus on reporting what you found. Move any interpretations / recommendations to the discussion.	We have rephrased the sentence on stigma and removed the section on the importance of talking about differences, as this was more a discussion point following from the data, than data itself.

Page 10<br>Page 39: Be careful not to put 'discussion points' in your data sections - it happens here and there. Focus on reporting what the data showed you. Leave the interpretations for the discussion.	Following your suggestions and comments, as well as comments by reviewer 1, we have gone through the results section and have rephrased sentences to clarify it followed from our findings or have removed sentences that contained interpretation or discussion of findings.
Page 11<br>Line 17: This sounds more like a discussion / introduction to this topic- stick with what the data shows. You can add this to discussion later.	We have rewritten the sentence, focussing on what was discussed during focus groups, and have removed any introduction and discussion of the findings.
Page 12:<br>Line 3: Rephrase to show that this is what the data showed (like the previous paragraph)	We have rephrased this sentence to highlight this is what the data showed.
Page 13:<br>Line 23 Make sure your paragraph reflect the data. If this is what your participants said -<br>then make that clear.<br>Line 46L Great discussion and reflection. If there are discussion points that I flagged in your results, you can add these in if it feels important.	We have rephrased the sentence so it is clear that this is what the data showed.

VERSION 2 - REVIEW

Reviewer 1

Name Vesentini, Lara

Affiliation Vrije Universiteit Brussel, Mental Health and Wellbeing Research Group

Date 07-Nov-2025

COI

Thank you for this valuable and nice work.
